# Supplementary figures and images for: zfh2 controls progenitor cell activation and differentiation in the adult Drosophila intestinal absorptive lineage
Source: PLoS Genet. 2019 Dec 16;15(12):e1008553. doi: 10.1371/journal.pgen.1008553 (PMC6936859; doi:10.1371/journal.pgen.1008553)

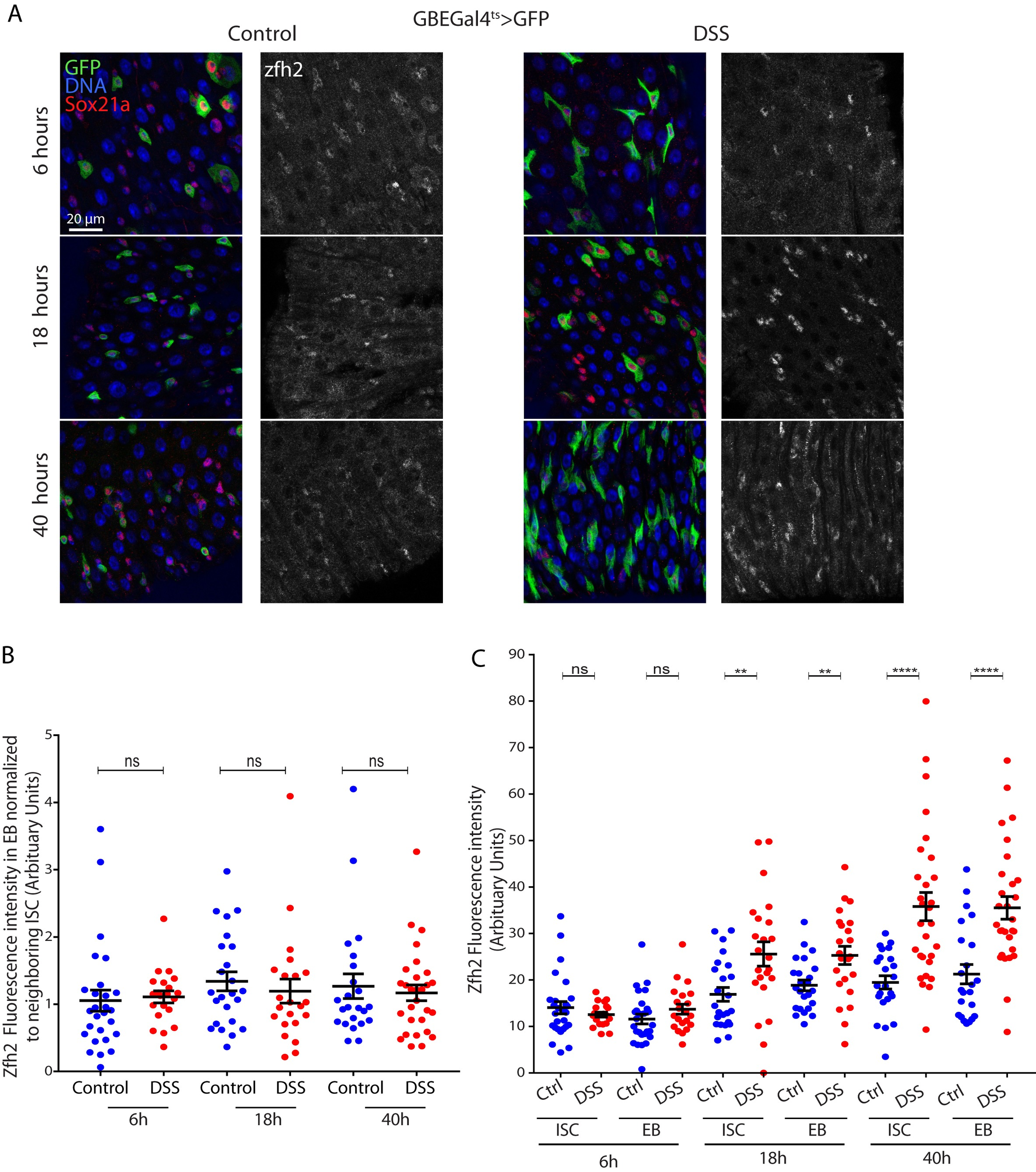

Supplement: S1 Fig — (A) Representative confocal image of the posterior midgut. EB are labeled by GBEGal4ts>mCD8GFP. Sox21a and zfh2 are detected via immunohistochemistry in ISC and EB. Flies are fed either DSS or Sucrose for 6, 18 or 40 hours before dissection and fixation. zfh2 protein is detected via immunohistochemistry. (B) zfh2 protein levels in EB are measured by quantification of zfh2 fluorescence in individual cells. Values are normalized to neighboring ISC. zfh2 protein is expressed at similar levels in both ISC and EB. (C) zfh2 protein levels in EB and ISC are measured by quantification of zfh2 fluorescence in individual cells. zfh2 protein levels increase after DSS mediated stress. In B and C, values are presented as average +/- s.e.m, and p-values are calculated using a two-tailed Student’s t-test. (TIF) [file pgen.1008553.s001.tif]

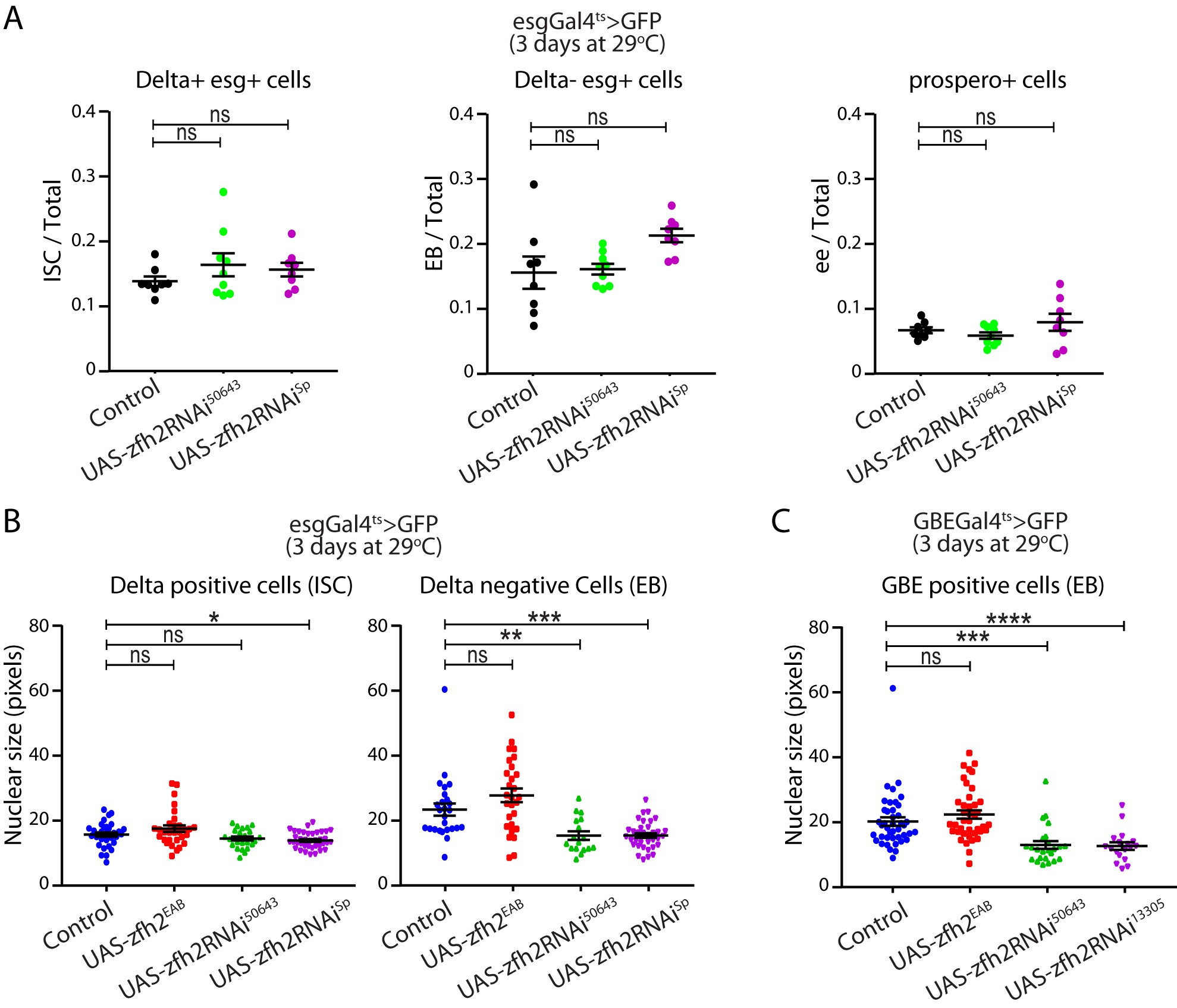

Supplement: S2 Fig — (A) ISC and EB are labeled by esgGal4ts>GFP. ISC and enteroendocrine cells are labeled via immunohistochemistry against delta and prospero respectively. zfh2 is knocked-down by driving dsRNA against zfh2 using esgGal4ts. Number of ISC (GFP+,Delta+), EB (GFP+,Delta-) and ee (Prospero+) cells are quantified and normalized to the total number of cells per ROI. Each value represents a ROI. (B) ISC and EB are labeled by esgGal4ts > GFP. ISC and enteroendocrine cells are labeled via immunohistochemistry against delta and prospero respectively. zfh2 is over-expressed by driving the UAS-zfh2EAB transgene using esgGal4ts. zfh2 is knocked-down by driving dsRNA against zfh2 using esgGal4ts. Nuclear size of ISCs and EBs are quantified by measuring nuclear area of individual cells. zfh2 knock down via dsRNA blocks endoreplication in EBs. (C) EB are labeled by GBEGal4ts>mCD8GFP. zfh2 is over-expressed by driving the UAS-zfh2EAB transgene using GBEGal4ts. zfh2 is knocked-down by driving dsRNA against zfh2 using GBEGal4ts. Nuclear size of EBs are quantified by measuring nuclear area of individual cells. zfh2 knock down via dsRNA blocks endoreplication in EBs. In A, B and C, values are presented as average +/- s.e.m, and p-values are calculated using a two-tailed Student’s t-test. (TIF) [file pgen.1008553.s002.tif]

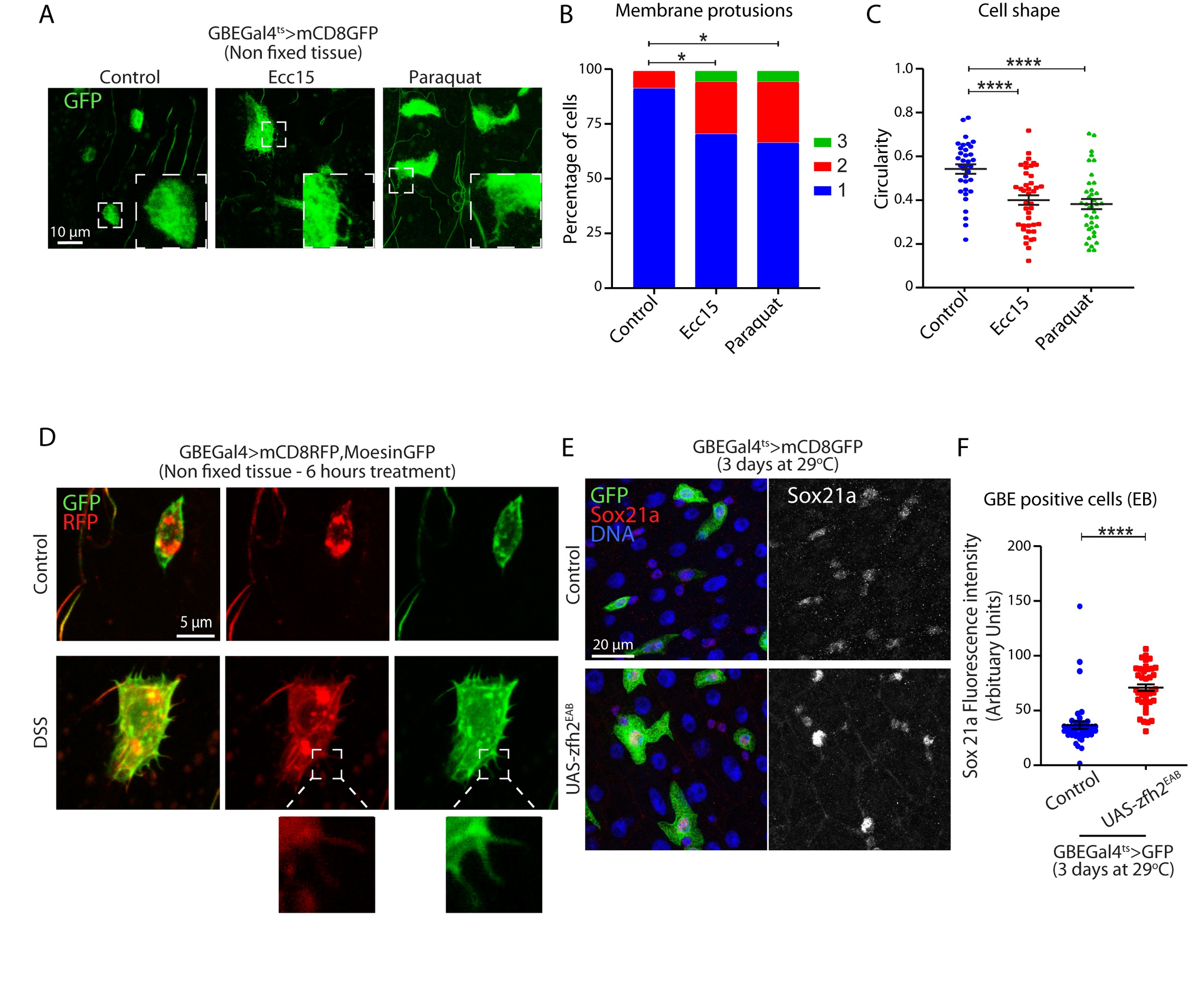

Supplement: S3 Fig — (A) Representative confocal images of non-fixed posterior midguts. EB are labeled by GBEGal4>mcD8GFP. Stress mediated EB activation is induced by feeding flies Paraquat or Ecc15 for 3–4 hours. Paraquat and ECC15 mediated stress is sufficient to increase the number of EBs with membrane protrusions (B) and decrease circularity (C). (D) Representative confocal images of non-fixed posterior midguts. EBs are labeled by GBEGal4>mcD8RFP, actin is labeled by GBEGal4>Moesin-GFP. Stress mediated EB activation is induced by DSS for 6 hours. Membrane protrusions contain actin. (E) Representative confocal images of posterior midguts. EB are labeled by GBEGal4ts>GFP. zfh2 is over-expressed by driving the UAS-zfh2EAB transgene using GBEGal4ts. Sox21a is detected via immunohistochemistry. (F) Quantification of sox21a protein levels in EB by quantifying mean sox21a fluorescence levels in individual cells. zfh2 over-expression in EB increases sox21a levels. In C and F values are presented as average +/- s.e.m, and p-values are calculated using a two-tailed Student’s t-test. In B p-values are calculated using the Mann-Whitney test. (TIF) [file pgen.1008553.s003.tif]

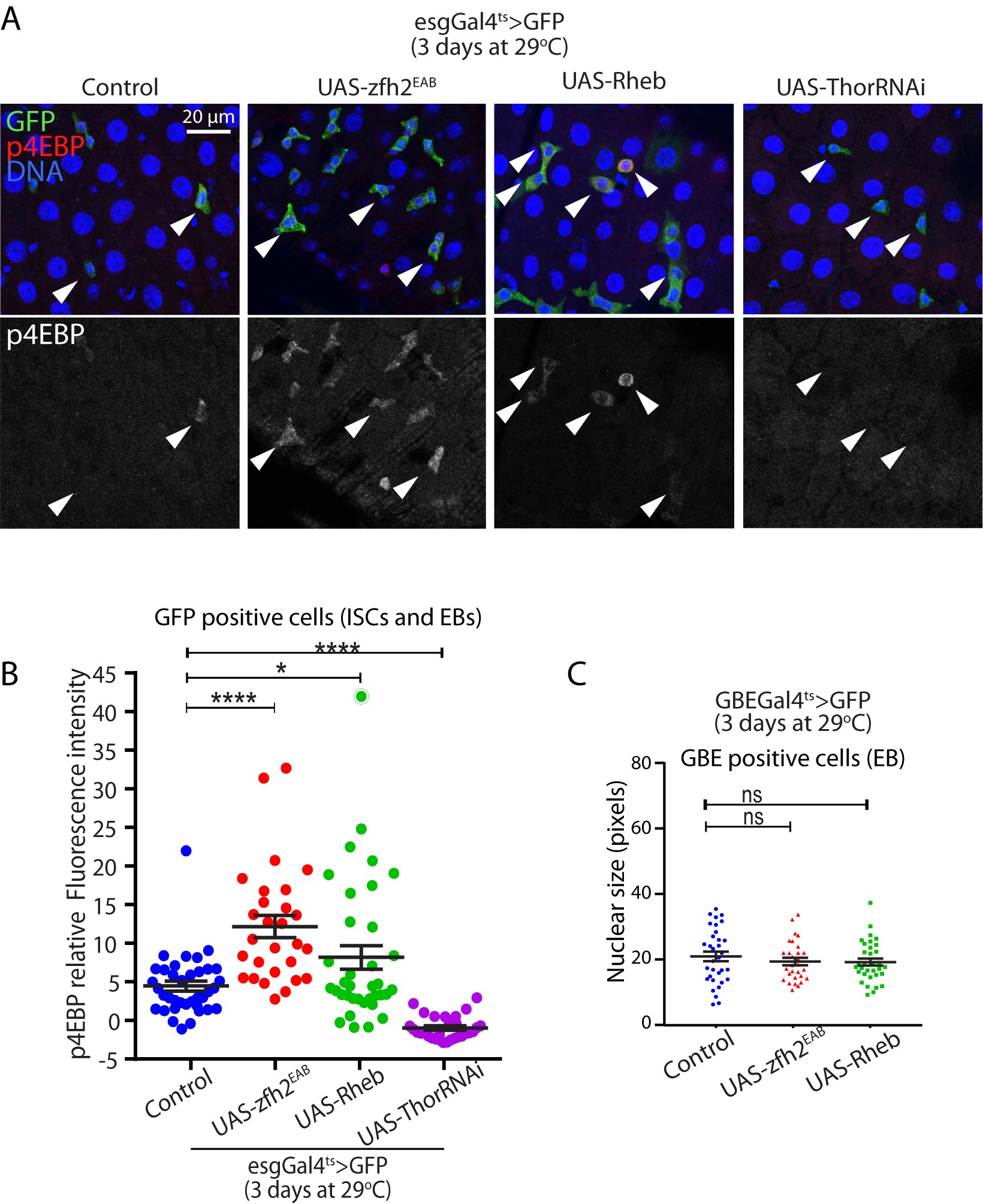

Supplement: S4 Fig — (A) zfh2 is over-expressed by driving the UAS-zfh2EAB transgene using esgGal4 ts. 4EBP (Thor) is knocked-down in EB by driving dsRNA using EsgGal4ts. Tor activity is stimulated by over-expression of the Tor activator Rheb. p4EBP is labeled via immunohistochemistry. (B) Protein levels are quantified by measuring mean fluorescence intensity of individual cells. Inducing EB activation via zfh2 over-expression is sufficient to increase Tor signaling activity. (C) EB are labeled by GBEGal4ts > mcD8GFP. zfh2 is over-expressed by driving UAS-zfh2EAB using GBEGal4ts. Tor activity is induced by over-expressing Rheb using GBEGal4ts. Nuclear size of EB are quantified by measuring nuclear area of individual cells. In B and C values are presented as average +/- s.e.m, and p-values are calculated using a two-tailed Student’s t-test. (TIF) [file pgen.1008553.s004.tif]

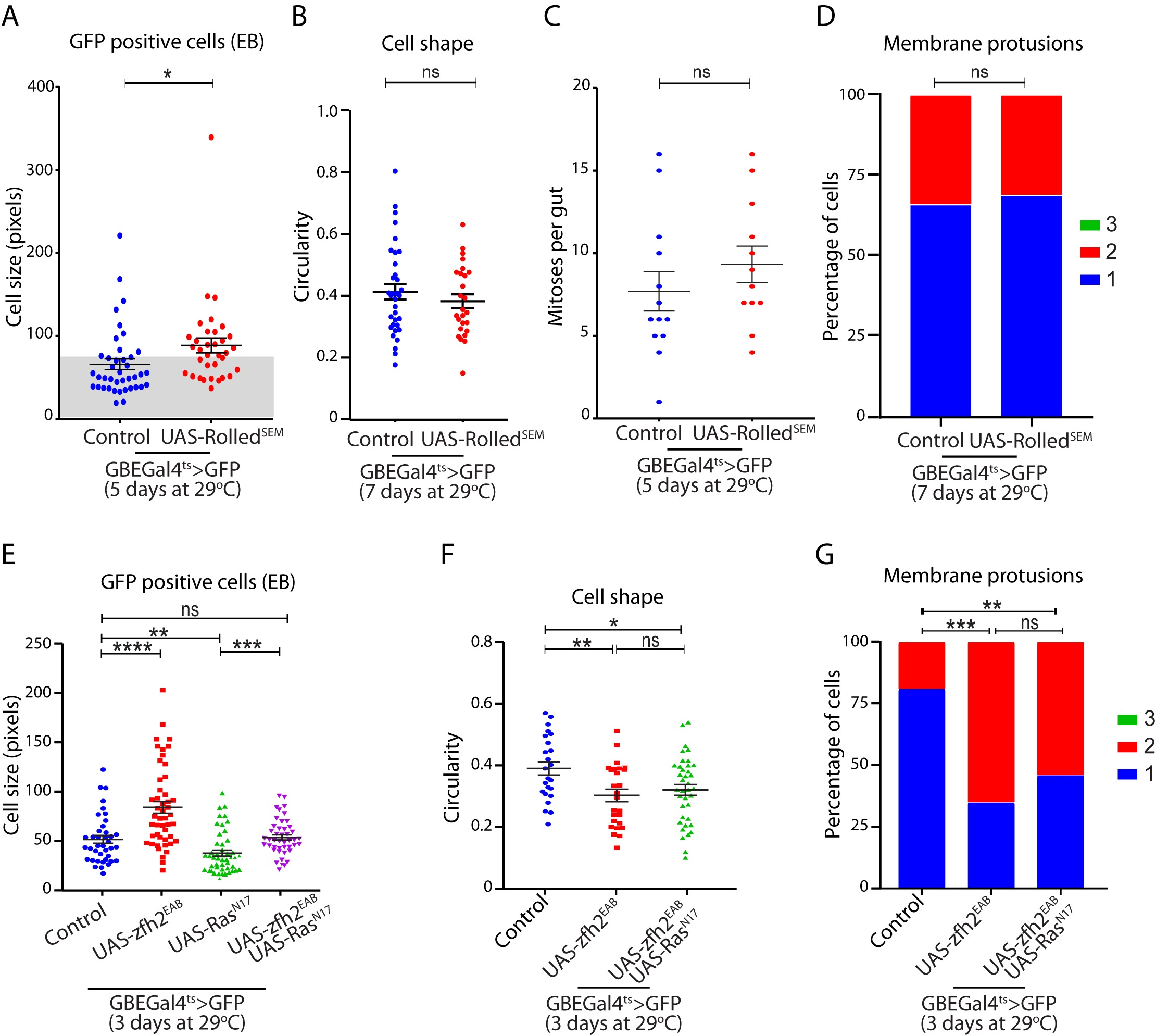

Supplement: S5 Fig — (A,B,C,D) ERK activity is induced in EB by driving the expression of the activated form of ERK (RolledSEM) using GBEGal4ts. EB are labeled by GBEGal4ts> mCD8GFP. (A) Cell size of EB are quantified by measuring cell area of individual cells. ERK activity induces EB growth. Inducing ERK activity is not sufficient to induce changes in cell morphology, measured by cell circularity (B), an increase on mitoses per gut, detected via immunohistochemistry against phosphoHistone H3 (C), or formation of membrane protrusions (D). (E,F,G) Ras activity is blocked in EB by driving expression of the dominant negative form of Ras (RasN17) using GBEGal4ts. zfh2 is over-expressed by driving the zfh2EAB transgene using GBEGal4ts. (E) Cell size of EB are quantified by measuring cell area of individual cells. Blocking Ras activity blocks EB growth cell-autonomously. Inducing EB activation induces growth in RasN17 EB. Blocking Ras activity is not sufficient to block changes in cell morphology, measured by cell circularity (F) or formation of membrane protrusions (G) associated with zfh2 mediated EB activation. In A, B, C, E, F values are presented as average +/- s.e.m, and p-values are calculated using a two-tailed Student’s t-test. In D, G p-values are calculated using the Mann-Whitney test. (TIF) [file pgen.1008553.s005.tif]

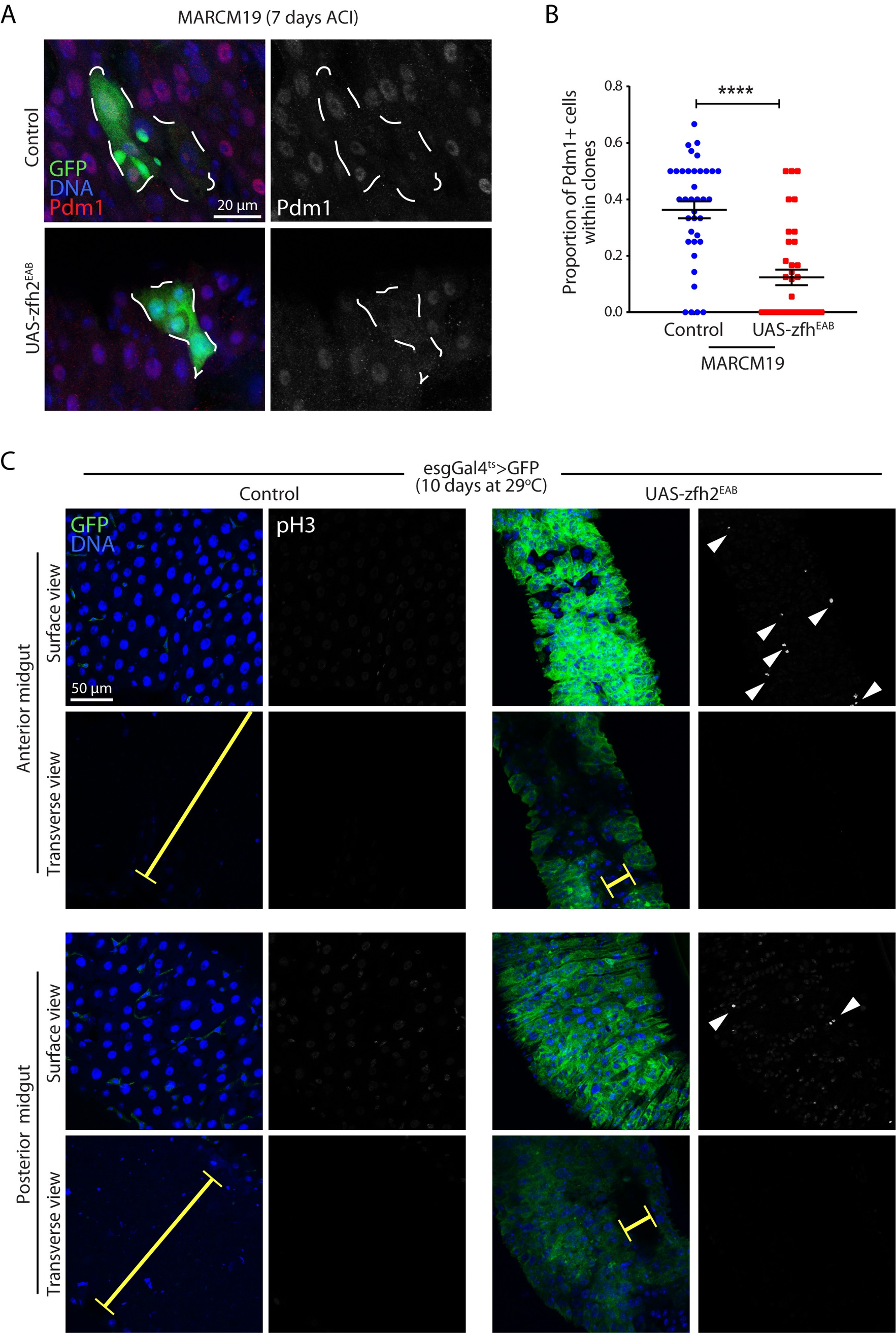

Supplement: S6 Fig — (A) Representative confocal images of control and zfh2 over-expressing MARCM clones in the posterior midgut, 7 days after induction. ECs are labeled via immunohistochemistry against Pdm1. (B) Proportion of EC per clone is quantified showing that UAS-zfh2 EAB clones contain a significantly reduced the number of EC compared to controls. (C) Representative confocal images of the posterior and anterior midgut. zfh2 is over-expressed by driving UAS-zfh2EAB using esgGal4ts. Mitotic cells are identified via immunohistochemistry against phosphoHistone H3 (arrowhead). Long term zfh2 over-expression leads to multilayered escargot expressing tumors in both the posterior and the anterior midgut. A high number of mitotically active cells are present in the basal layer of the tumor. Gut lumen is indicated by yellow brackets in the transverse views. In B values are presented as average +/- s.e.m, and p-values are calculated using a two-tailed Student’s t-test. (TIF) [file pgen.1008553.s006.tif]
