# Supplementary material for: zfh2 controls progenitor cell activation and differentiation in the adult Drosophila intestinal absorptive lineage
Source: PLoS Genet. 2019 Dec 16;15(12):e1008553. doi: 10.1371/journal.pgen.1008553 (PMC6936859; doi:10.1371/journal.pgen.1008553)
Supplement: S1 File — For each figure (main figures and supplementary figures), the genotypes of all the animals used in the described experiments is detailed here. (DOCX) [file pgen.1008553.s007.docx]

**S1 File. Detailed genotypes of animals used in this study.**

**Figure 1**

(A) esgGal4, UAS-GFP/+; TubG80^ts^/+

esgGal4, UAS-GFP/+; TubG80^ts^/ UAS-zfh2RNAi^13305^

esgGal4, UAS-GFP/+; TubG80^ts^/ UAS-zfh2RNAi^50643^

(B) esgLacz/UAS-GFP; zfh2Gal4^GMR73G11^/+

esgLacz/UAS-GFP;; zfh2Gal4^LP40^/+

**Figure 2**

(A, B) esgGal4, UAS-GFP/+; TubG80^ts^/+

esgGal4, UAS-GFP/Cyo; TubG80^ts^/+; UAS-zfh2^EAB^/+

esgGal4, UAS-GFP/+; TubG80^ts^/ UAS-zfh2RNAi^50643^

esgGal4, UAS-GFP/+; TubG80^ts^/ UAS-zfh2RNAi^Sp^

(C, D) GBEGal4, UAS-mCD8GFP/+; TubG80^ts^/+

GBEGal4, UAS- mCD8GFP/Cyo; TubG80^ts^/+; UAS-zfh2^EAB^ /+

GBEGal4, UAS- mCD8GFP/+; TubG80^ts^/ UAS-zfh2RNAi^50643^

GBEGal4, UAS- mCD8GFP/+; TubG80^ts^/ UAS-zfh2RNAi^13305^

**Figure 3**

(A, B,C) GBEGal4,UAS-mCD8GFP/+; TubG80^ts^/+

(D, E, F) GBEGal4, UAS-mCD8GFP/+; TubG80^ts^/+

GBEGal4, UAS- mCD8GFP/+; TubG80^ts^/ UAS-zfh2RNAi^13305^

GBEGal4, UAS- mCD8GFP/+; TubG80^ts^/ UAS-zfh2RNAi^50643^

(G, H, I) GBEGal4, UAS-mCD8GFP/+; TubG80^ts^/+

GBEGal4, UAS- mCD8GFP/Cyo; TubG80^ts^/+; UAS-zfh2^EAB^ /+

**Figure 4**

(A) GBEGal4, UAS- mCD8GFP /UAS-RedStinger,UAS-FLP,UbiFRTStopFRTStinger; TubG80^ts^/+

GBEGal4, UAS- mCD8GFP /UAS-RedStinger,UAS-FLP,UbiFRTStopFRTStinger; TubG80^ts^/ UAS-zfh2RNAi^50643^

(B) GBEGal4, UAS-mCD8GFP/+; TubG80^ts^/+

GBEGal4, UAS- mCD8GFP/+; TubG80^ts^/ UAS-zfh2RNAi^13305^

GBEGal4, UAS- mCD8GFP/+; TubG80^ts^/ UAS-zfh2RNAi^50643^

GBEGal4, UAS- mCD8GFP/+; TubG80^ts^/ UAS-zfh2RNAi^Sp^

(C) GBEGal4, UAS-mCD8GFP/+; TubG80^ts^/+

GBEGal4, UAS- mCD8GFP/Cyo; TubG80^ts^/+; UAS-zfh2^EAB^ /+

DeltaGal4,TubG80 ^ts^/+;

DeltaGal4,TubG80 ^ts^/Cyo; UAS-zfh2^EAB^ /+

esgGal4, UAS-EYFP/+; Su(H)-Gal80, TubGal80 ^ts^ /+

esgGal4, UAS-EYFP/Cyo; Su(H)-Gal80, TubGal80 ^ts^ /+; UAS-zfh2^EAB^ /+

**Figure 5**

(A) GBEGal4, UAS-mCD8GFP/+; TubG80^ts^/+

GBEGal4, UAS- mCD8GFP/Cyo; TubG80^ts^/+; UAS-zfh2^EAB^ /+

GBEGal4, UAS-mCD8GFP/UAS-ThorRNAi; TubG80^ts^/+

(B) GBEGal4, UAS-mCD8GFP/+; TubG80^ts^/+

GBEGal4, UAS- mCD8GFP/Cyo; TubG80^ts^/+; UAS-zfh2^EAB^ /+

GBEGal4, UAS- mCD8GFP/+; TubG80^ts^/UAS-TSC1+2

GBEGal4, UAS- mCD8GFP/+; TubG80^ts^/UAS-TSC1+2; UAS-zfh2^EAB^ /+

(C) GBEGal4, UAS-mCD8GFP/+; TubG80^ts^/+

GBEGal4, UAS- mCD8GFP/UAS-Rheb; TubG80^ts^/+

GBEGal4, UAS- mCD8GFP/UAS-Rheb; TubG80^ts^/ UAS-zfh2RNAi^13305^

GBEGal4, UAS- mCD8GFP/UAS-Rheb; TubG80^ts^/ UAS-zfh2RNAi^50643^

(D, E, F) GBEGal4, UAS-mCD8GFP/+; TubG80^ts^/+

GBEGal4, UAS- mCD8GFP/UAS-Rheb; TubG80^ts^/+

**Figure 6**

(A, B) GBEGal4, UAS-mCD8GFP/+; TubG80^ts^/+

GBEGal4, UAS- mCD8GFP/UAS-InR ^Act^; TubG80^ts^/+

GBEGal4, UAS- mCD8GFP/ UAS-InR ^Act^; TubG80^ts^/ UAS-zfh2RNAi^13305^

(C, D) GBEGal4, UAS-mCD8GFP/+; TubG80^ts^/+

GBEGal4, UAS- mCD8GFP/UAS-InR ^Act^; TubG80^ts^/+

(E, F) GBEGal4, UAS-mCD8GFP/+; TubG80^ts^/+

GBEGal4, UAS- mCD8GFP/Cyo; TubG80^ts^/+; UAS-zfh2^EAB^ /+

GBEGal4, UAS- mCD8GFP/UAS-InR^DN^; TubG80^ts^/+

GBEGal4, UAS- mCD8GFP/UAS-InR^DN^; TubG80^ts^/+; UAS-zfh2^EAB^ /+

**Figure 7**

(A, B, C, D) EsgGal4, UAS-GFP/+; TubG80^ts^/+

esgGal4, UAS-GFP/Cyo; TubG80^ts^/+; UAS-zfh2^EAB^ /+

(E) GBELacZ/+; EsgGal4, UAS-GFP/+; TubG80^ts^/+

GBELacZ/+; EsgGal4, UAS-GFP/Cyo; TubG80^ts^/+; UAS-zfh2^EAB^ /+

(F) GBEGal4, UAS-mCD8GFP/+; TubG80^ts^/+

GBEGal4, UAS- mCD8GFP/Cyo; TubG80^ts^/+; UAS-zfh2^EAB^ /+

**Supplementary Figure 1**

(A, B, C) GBEGal4, UAS-mCD8GFP/+; TubG80^ts^/+

**Supplementary Figure 2**

(A) esgGal4, UAS-GFP/+; TubG80^ts^/+

esgGal4, UAS-GFP/+; TubG80^ts^/UAS-zfh2RNAi^50643^

esgGal4, UAS-GFP/+; TubG80^ts^/UAS-zfh2RNAi^Sp^

(B) esgGal4, UAS-GFP/+; TubG80^ts^/+

esgGal4, UAS-GFP/Cyo; TubG80^ts^/+; UAS-zfh2^EAB^/+

esgGal4, UAS-GFP/+; TubG80^ts^/UAS-zfh2RNAi^50643^

esgGal4, UAS-GFP/+; TubG80^ts^/UAS-zfh2RNAi^Sp^

(C) GBEGal4, UAS-mCD8GFP/+; TubG80^ts^/+

GBEGal4, UAS- mCD8GFP/Cyo; TubG80^ts^/+; UAS-zfh2^EAB^/+

GBEGal4, UAS- mCD8GFP/+; TubG80^ts^/UAS-zfh2RNAi^50643^

GBEGal4, UAS- mCD8GFP/+; TubG80^ts^/UAS-zfh2RNAi^13305^

**Supplementary Figure 3**

(A, B, C) GBEGal4,UAS-mCD8GFP/+;TubG80 ^ts^/+

(D) GBEGal4, UAS-mCD8RFP/UAS-MoesinGFP

(E, F) GBEGal4, UAS- mCD8GFP/+; TubG80^ts^/+

GBEGal4, UAS- mCD8GFP/Cyo; TubG80^ts^/+; UAS-zfh2^EAB^/+

**Supplementary Figure 4**

(A, B) esgGal4, UAS-GFP/+; TubG80^ts^/+

esgGal4, UAS-GFP/Cyo; TubG80^ts^/+; UAS-zfh2^EAB^ /+

esgGal4, UAS-GFP/ UAS-Rheb; TubG80^ts^/+

esgGal4, UAS-GFP/ UAS-ThorRNAi; TubG80^ts^/+

(C) GBEGal4, UAS-mCD8GF/+; TubG80^ts^/+

GBEGal4, UAS-mCD8GFP/Cyo; TubG80^ts^/+; UAS-zfh2^EAB^ /+

GBEGal4, UAS-mCD8GFP/UAS-Rheb; TubG80^ts^/+

**Supplementary Figure 5**

(A, B, C, D) GBEGal4, UAS- mCD8GFP/+; TubG80^ts^/+

GBEGal4, UAS- mCD8GFP/UAS-Rolled^SEM^; TubG80^ts^/+

(E) GBEGal4, UAS- mCD8GFP, TubG80^ts^/Cyo

GBEGal4, UAS- mCD8GFP, TubG80^ts^/Cyo; ; UAS-zfh2^EAB^ /+

GBEGal4, UAS- mCD8GFP, TubG80^ts^/UAS-RasN17

GBEGal4, UAS- mCD8GFP, TubG80^ts^/UAS-RasN17; ; UAS-zfh2^EAB^ /+

(F, G) GBEGal4, UAS- mCD8GFP, TubG80^ts^/Cyo

GBEGal4, UAS- mCD8GFP, TubG80^ts^/Cyo; ; UAS-zfh2^EAB^ /+

GBEGal4, UAS- mCD8GFP, TubG80^ts^/UAS-RasN17; ; UAS-zfh2^EAB^ /+

**Supplementary Figure 6**

(A, B) hsFLP, tubGal80, FRT19A/FRT19A; TubGal4, UAS-GFP

hsFLP, tubGal80, FRT19A/FRT19A; TubGal4, UAS-GFP;; UAS-zfh2^EAB^ /+

(C) esgGal4, UAS-GFP/+; TubG80^ts^/+

esgGal4, UAS-GFP/Cyo; TubG80^ts^/+; UAS-zfh2^EAB^ /+
