# Supplementary material for: zfh2 controls progenitor cell activation and differentiation in the adult Drosophila intestinal absorptive lineage
Source: PLoS Genet. 2019 Dec 16;15(12):e1008553. doi: 10.1371/journal.pgen.1008553 (PMC6936859; doi:10.1371/journal.pgen.1008553)
Supplement: Attachment — Submitted filename: Response to Reviewers.docx [file pgen.1008553.s010.docx]

**Response to Reviewers**

First, we would like to thank the reviewers for their constructive comments on our manuscript. We believe that the suggested revisions and additions significantly improved the quality of our study. We are addressing all the concerns in detail below.

The main points are:

- We provide additional data and experimental controls to further support our model that Zfh2-mediated activation can be separated from growth signals (TOR and Ras/MAPK signaling). This includes additional analysis of TOR activity in situ in response to Zfh2 and genetic interactions between Zfh2 and Ras.
- We show that EB activation can be detected in response to all stressors tested, using the methodology established in this study.
- We further refine our description of the tumors mediated by Zfh2 over-expression in the revised version.
- We edited our manuscript, figures and figure legends to clarify aspects that were mentioned as unclear by the reviewers.
- We include two supplementary data files that contains all the numerical values associated with the main figures and supplementary figures.

**Reviewer #1: The authors report here that Zfh2 is an ISC/EB specific factor that regulates EB activation and differentiation to ECs. Furthermore, they report that Zfh2 is required and sufficient to drive activation of EBs. This includes cell shape change, cell size increase, transient formation of thin membrane protrusions and increased compensatory ISC proliferation. Additionally, they report that Zfh2 is upstream of TOR and MAPK and parallel to insulin signaling and that Zfh2 overexpression results in dysplastic lesions.

Overall this study explores an important question about the early stages of differentiation of intestinal progenitors- a process that is not well-understood. The data provided about Zfh2’s role in EB activation (i.e., cell shape and size change, protrusion formation, requirement for differentiation) are convincing; however, the mechanism(s) into how Zfh2 is regulated or regulates EB activation/differentiation needs to be strengthened. The claim that Zfh2 is upstream of TOR and MAPK and parallel to insulin signaling is not well supported with the data provided.

I have outlined my concerns below:

1. The role of Zfh2 needs to be put into context with other pathways known to regulate EB growth and differentiation, e.g. Notch or JAK-STAT signaling. The authors describe in the introduction that Notch controls Zfh2 expression in developing fly wing discs (page 6). Does the Notch signal in EBs promote Zfh2 expression or activity in EBs? This is important since the authors report that Zfh2 is required in EBs that have received a Notch signal (GBE+) for their growth and differentiation to ECs.**

We agree that understanding the potential connection between Notch and zfh2 in EBs in important for our study. However, we have no data to support the notion that zfh2 is controlled by or controls Notch in the intestine. For example, while Notch signaling is inactive in ISCs and active in EBs, zfh2 expression is similar in ISCs and EBs (Figure 1A, S1); we mention this interpretation in the revised version on page 7. Also, the zfh2 protein is detected mostly detected in the nuclei of both cells (Figure S1). Reciprocally, we found no evidence that the activity of the Su(H)GBEGal4 or Su(H)GBELacZ Notch reporters are affected when zfh2 expression is manipulated.

In order to directly address this question, we planned on performing the experiment suggested here. Unfortunately, leakiness of the GBEGal4ts driver prevented us from completing the experiment: GBEGal4ts>UAS-NotchINTRA animals die at pupal stages (like GBEGal4ts>UAS-RasV12 animals do), even when grown at temperature as low as 16°C.

**2. The study nicely tries to establish a standard to study EB activation and uses DSS stress to induce EB activation. It is important though to demonstrate that these activation events (i.e., protrusion formation) occur upon a variety of stresses shown to promote ISC proliferation (and thus likely increase activated EB numbers), e.g. pathogen infection, oxidative stress, DNA damage. Are these morphological changes common features of activation in other stress conditions or rather only a response to DSS treatment which affects the extracellular matrix. Antonello et al., 2015 reported EB protrusion formation only under non-stress conditions. Do EBs differentiating into EEs also form protrusions? Presumably, they also should.**

We agree that it is important to test whether EB activation is part of the regenerative process, irrespective of the stimulus. We used the methodology described in this study to investigate EB activation in response to infection and oxidative stress. We now show in Figure S3A-C that EB activation can be detected 3-4hours after feeding flies an Ecc15 bacterial culture or Paraquat solution. Significant increase in protrusion formation and decreased circularity can be measured in EBs, 3 to 4 hours following both treatments. This experiment is now mention on page 10 of the revised manuscript.

It would be interesting to investigate the cell morphological changes induced and required during EE differentiation. However, we and others reported that EEs typically differentiate from progenitors that are distinct from EBs (ie pre-EEs). It is also worth noting that because of their difference in cell size, shape and localization in the pseudo-epithelium, pre-EEs and EBs may have very different differentiation requirements regarding cell-cell interactions and cell migration. For all these reasons, we limit the current study to the absorptive lineage and EBs.

**3. The authors show that Zfh2 is required for cell size increase but is not sufficient to promote endoreplication. This data is confounding since the authors claim that Zfh2 also promotes TOR signaling which has been shown to promote endoreplication in fly tissues (Zielke et al., 2011).**

While we agree that activation of TOR signaling in some fly tissues may be sufficient to induce endoreplication, we did not confirm this observation in enteroblasts. Like for Zfh2, over-expression of Rheb for 3days using the Su(H)GBEGal4ts driver leads to a significant increase in cell size (Figure5C) but does not result in a detectable increase in nuclear size. This suggests that, in these cells, cell growth can be separated from nuclear growth (and presumably endoreplication).

These data have been included in the revised version (Figure S4C) and mentioned on page 13. These support our conclusion that Zfh2-mediated EB activation is required for ToR-mediated cell growth.

**4. In Figure 3D, why are there large protrusions observed in the control (without DSS)?**

We apologize for the misperception here, as there are no large protrusions in control conditions. We believe that what is referred to here is the autofluorescence signal from trachea that we observe in some of our images of live tissues.

To avoid any confusion, we updated this figure with higher magnification inserts that focus on the cell membrane and illustrate the absence of protrusions. We also mention the presence of signal from the trachea in the legend of this figure.

**5. In Figure 4A, the overlap of Pdm1 with GBE>trace is difficult to observe. A separation of fields will help here. Is Zfh2 required for EE differentiation?**

We agree that merge only images made it difficult to see the Pdm1 staining signal. We have updated this figure: We now show the Pdm1 channel individually; we also added arrowheads to point to GFP-positive Pdm1-positive newly differentiated ECs in stressed controls as well as asterisks to indicate Pdm1-negative EBs (in unstressed conditions and Zfh2 loss-of-function).

Regarding EE differentiation, in these experiments we analyzed the lineage of EBs specifically using the GBEGal4ts>G-TRACE lineage tracing. As GBEGal4-positive EBs do not typically give rise to EEs, this assay does not allow us to investigate Zfh2 requirement for EE differentiation.

**6. Do Zfh2 levels affect E-Cadherin levels in EBs? The text on page 23 states that Zfh2 can induce DE-Cadherin degradation, but this data is not in the manuscript.**

We believe that the reviewer refers here to a sentence in the discussion on Page 19. We discuss in this paragraph the interaction between InR and zfh2, in the context of non-cell autonomous ISC proliferation induced by genetic manipulation in EBs. In the case of InR, it has been established that this phenomenon is mediated by a degradation of Ecadherin (Choi et al. 2011), leading us to mention the possibility that a similar mechanism may be induced downstream of zfh2 activity. We apologize if our original wording was confusing and we edited this part of our discussion to make it more evident that this is only a hypothesis at this point of our studies.

**7. The data shown to support TOR activation by Zfh2 is not yet convincing for the following reasons: 1) the phospho-4EBP antibody in Fig. 5A needs to be validated, preferably in situ (e.g., knockdown of 4EBP, overexpression of Rheb); 2) in Fig. 5B, Zfh2 overexpression increases phospho-4EBP in all esg+ cells (which presumably includes ISCs) in the field, yet there is no effect on ISC size or proliferation (Fig. 4C). This is surprising since it has been reported that activated ISCs can increase in size (Jiang et al., 2009); 3) in Fig. 5B, the activation of TOR signaling by Zfh2 overexpression is not terribly convincing, particularly since the background in the field shown is increased too. Furthermore, since Zfh2 overexpressing EBs are larger, the quantitation data needs to be normalized to cell volume; 4) the variation in phospho-4EBP levels on the western blots in Fig. S4A after Rheb overexpression is concerning as Rheb overexpression should give a consistent increase in TOR signaling. To support this it would be helpful to see phospho-4EBP levels in EBs overexpressing Rheb. For these reasons, it is not clear whether Zfh2 activates TOR signaling. Since TOR signaling is an important regulator of protein translation, cell growth and other cellular processes, it is also not terribly surprising that inhibiting TOR signaling blocks Zfh2 induced EB growth (Fig. 5C).** **Does Zfh2 loss in EBs increase TSC2 levels? (see Kapuria et al., 2012).**

We agree here that demonstrating a significant and specific induction of Tor signaling in EBs in response to zfh2 over-expression is critical to interpret the genetic interactions between these two pathways. To this end, we performed a series of new experiments using immunostaining and including the suggested experimental controls. We now show, using the esgGal4 driver, that increased phospho4EBP signal is detected when Tor is activated by expressing UAS-Rheb and that this signal is lost when Thor/4EBP expression is inhibited by RNAi (Figure S4A). This supports the differences observed by western blot on total intestinal protein extracts (Note that the low levels detected in control samples do not allow us to reliably measure a loss in ThorRNAi expressing intestines using this assay). We also show that the p4EBP signal is specifically lost in EBS when Thor/4EBP expression is inhibited by RNAi using the GBEGal4ts driver (Figure 5A).

Regarding the method used to measure fluorescence intensity, we have now described in more detail our method in the appropriate section. Importantly, these data are presented as average pixel intensity within GFP-positive cells, normalized to average background pixel signal (ROI selected between GFP-positive cells). Therefore, the differences reported do not depend on cell size or cell volumes.

We believe that our p4EBP quantifications and experimental controls convincingly demonstrate that over-expressing zfh2 is sufficient to increase Tor activity in EBs.

Regarding the absence of effect on ISC size and proliferation, our interpretation here is that the moderate increase in Tor activity include by zfh2 over-expression is not sufficient to affect ISCs, as opposed to EBs that are primed to grow in response to differentiation signals. It is possible that more manipulations that more severely increase Tor activity may result in ISC growth and proliferation. Overall, we do not believe that our results conflict with previous studies but demonstrate that zfh2-mediated activation is required for Tor-mediated cell growth.

**8. The abstract claims that Zfh2 is upstream of MAPK, but there is no data in the manuscript to support this conclusion. MAPK activity has been observed in ISCs (Jiang et al. 2011; Biteau and Jasper, 2011; Liang et al., 2017) but not EBs. The authors should show that MAPK activity is present in differentiating EBs under non-stress conditions. If so, is Zfh2 required for MAPK activation in EBs? Is MAPK (Rolled) required for Zfh2 to promote EB activation and differentiation?**

This is an important point and we now provide more genetic evidence to support our conclusion. We previously showed that activation of MAPK, by the expression of RolledSEM (gain-of-function allele of Rolled/ERK) induces cell growth but is not sufficient to drive EB activation, as measured by the formation of membrane protrusions and decreased cell circularity (Figure S5A,S5B,S5C,S5D). But, we had not tested the requirement for the Ras/MAPK pathway for zfh2-mediated EB activation. We now provide these data. We show that strong inhibition of this pathway (by expressing the dominant negative form RasN17) is sufficient to reduce cell growth but does not affect the ability of zfh2 to induce EB activation (Figure S5E, S5F, S5G).

It is also important to note that under normal growth conditions, the vast majority of EBs are dormant, with low dpERK signals. However, upon induction of EB activation and growth by stress signals, high levels of dpERK can be detected in these EBs / early ECs (see examples in Jiang et al. 2011; Xiang et al. 2017). This supports a model where MAPK activation contributes to EB growth, at least under stress conditions.

Our data does not conflict with this model, and demonstrate the EB activation requires zfh2, but does not require Tor- or MAPK-mediated cell growth. Thus, our work places zfh2 upstream of these two pathways in the sequence of events leading to EB differentiation.

9. In Fig. 6A, the authors show that overexpression of the activated insulin receptor promotes growth in Zfh2-deficient EBs. But this does not necessarily suggest that insulin signaling acts parallel to Zfh2 in promoting EB differentiation. The loss of TOR from EB blocks its growth and differentiation (similar to Zfh2 loss), but this can be bypassed during regeneration through activation of EGFR signaling (Jiang et al., 2016).

Yes, our data shows that over-expression of activated InR promotes EB growth and non-cell autonomous ISC proliferation in the absence of zfh2 (Figure 6A). But, conversely, we also show that over-expression of zfh2 promotes EB growth and non-cell autonomous ISC proliferation in the absence of InR signaling (Zfh2+InRDN) (Figure 6F). Together, this strongly suggest to us that that InR and zfh2 control these aspects of early EB differentiation independently from one another, i.e in parallel. We clarified our conclusion at the end of the paragraph describing these data, on page 16, to explain our interpretation of our results.

**10. It’s not clear what Fig. 2A is showing even after reading the figure legend.**

We forgot to mention what the arrow heads and asterisks point to in the legend. These images illustrate how ISCs (GFP+Dl+ cells) and EBs (GFP+Dl- cells) are identified, in order to measure differences in cell size in zfh2 gain-of-function and loss-of-function. We updated the legend to make this more evident.

**Some general comments:

1. The title could be more specific and state “progenitor cell activation“**

Thank you for the suggestion. We agree that this makes the title more precise and we updated it.

**2. Drosophila should be capitalized and in italics throughout the text**

We have corrected this on the revised manuscript.

**3. The manuscript would benefit (if known) from more information in the introduction about zfh2 and its regulation/activity in a variety of systems**

We slightly expanded our introduction and discussion of zfh2 and its mammalian homologs (ATBF1/ZfHX3 and ZFH4). However, little is known about the mechanisms that control the activity of these transcription factors. For this reason, we believe that our description of the role of zfh2 in EB activation and differentiation will provide us with a valuable new model to study the function and regulation of these factors.

**Reviewer #2: In “Zfh2 controls cell activation and differentiation in the adult Drosophila intestinal lineage”, Villa, Meng and Biteau presents a very nice study of zfh2, a zinc-finger homeodomain transcription factor in regulating the activation of enteroblast (EB) in the adult fly. Under the physiological state, an EB can remain inactivated for up to 14days before differentiating into an enterocyte (EC). The molecular mechanism that confers EB with such long dormancy remains largely unexplored. Here Villa et al depicts zfh2 as a novel player in the determination of intestinal progenitor fate. By carefully tracking the morphological changes in EB coupled to loss- and gain-of-function approaches, the authors demonstrate a requirement for zfh2 for EB growth and activation induced by stress, yet maintaining ectopic zfh2 expression in activated EB blocks EB to EC differentiation, and eventually leads to tumor formation. Furthermore, by studying epistatic interaction between zfh and mTOR and insulin signaling, the authors are able to provide strong evidence that EB growth and morphological changes required for activation are largely separate processes.

While I like this study very much, I do have more questions regarding the data presented in Figure 7. The tumor phenotype of esg>zfh2 is convincing, striking and intriguing, and I would like to see a bit more characterization. First, are these tumors regionalized? For example, sox21a loss-of-function tumors preferentially grow in the anterior midgut (AMG) of the flies.**

The esgGal4ts>UAS-Zfh2 tumor phenotype are highly penetrant in both the anterior and the posterior midgut. We added images for both regions in Figure S6C. Of note, these tumors are much smaller in the copper cells region (mid midgut), where proliferation is generally reduced. These observations are now mentioned on page 15 and 16 of our revised manuscript.

**Secondly, is the tumor formation driven by proliferation from ISCs or elevated endoreplication of the EB cells? It would be a nice control to characterize the tumors further with PH3 staining and BrdU incorporation, while labeling Zhf2 overexpressing cells.**

We agree that this is an important point to support our model. We now show in Figure S6C that high numbers of pH3-positive cells can be detected in the multilayered tumor epithelia. This is limited to the very basal layers where we show that Delta+ ISCs reside. No pH3 positive cells can be detected in the more apical layers where arrested EBs are located. These results are now mentioned on page 16.

Of note, results of BrdU incorporation experiments would likely be very difficult to interpret as many of the arrested EBs that accumulate have nuclei larger than diploid cells, suggested that they undergo some level of endoreplication and would likely incorporate BrdU.

**Lastly, the high levels of accumulation of Sox21a in the tumors seems counterintuitive based on what we know. It is the current understanding that Sox21a is necessary for EB differentiation into ECs, and sufficient to drive the expression of Pdm1 in the ECs; Loss of Sox21a results in tumor formation, and re-expression of Sox21a specifically in EB cells can prevent this process. The authors here show that overexpression of Zfh2 is sufficient to induce Sox21a expression, yet it correlates with tumor formation. The authors resolve the discrepancy by claiming that zfh2 overexpression tumors and Sox21a loss of function tumors are different: “Sox21a-negative esg-positive early enteroblast in sox21a loss of function and Sox21A-high esg-positive late enteroblast in zfh2 gain of function”. This explanation is vague and the language is awkward. If the authors mean that the sox-21a accumulating tumors are late EB cells, is it possible to examine mir8-lacZ expression in this tumors? In addition to attributing the difference to “early” and late” EBs, is it conceivable that Zfh2 overexpression blocks Sox21a activity, and the target of Sox21a is engaged in an ectopic amplification loop of Sox21a expression? It would be interesting to know what happens to Sox21a expression in a zfh1 loss of function clone. Also, what happens to Zfh2 expression in a Sox21a loss of function tumor, and also assess if there is also loss of NRE marker expression in the most apical layer.**

We apologize for the lack of clarity in this part of our discussion and have rewritten and expanded this section on Page 21 to better explain the molecular and cellular differences between Sox21a-mutant and zfh2-overexpressing tumors.

We show that (i) zfh2-GOF tumors are Sox21-high, Sox21-LOF tumors are Sox21 null/low; (ii) zfh2-GOF tumors have lost Notch activity, Sox21-LOF tumors retain NRE activity; in our hands, zfh2-GOF tumors are more aggressive and prevalent that Sox21a-mutant tumors.

For these reasons, we believe that the most likely model is the one we discuss, where maintaining zfh2 expression arrests EB differentiation at a later stage than Sox21a-LOF.

We agree that this also suggest that maintaining zfh2, directly or indirectly, inhibits that ability of Sox21a to drive differentiation. We now mention this conclusion.

**Minor Issues:
1. The authors report an accumulation of EBs bearing small nuclei upon zfh2 knock-down. Based on this observation the authors propose that zfh2 is required for EB endoreplication. The authors should image endoreplication markers in WT and knock-down conditions to strengthen their conclusion.**

Our data show that EBs expressing zfh2RNAi are similar in cell size and nuclear size to ISCs (small and diploid). This demonstrates that these cells do not undergo endoreplication. Whether zfh2 directly or indirectly controls the endoreplication is unclear, although at this point we favor the model presented here where zfh2 is required for EB activation prior to growth and endoreplication, rather that directly controlling these processes. Answering this question would require the identification of zfh2 target genes that is beyond the scope of this manuscript.

Regarding the use of markers, BrdU or EdU incorporation is a well-accepted method to label endoreplicating cells. However, as mentioned above, results of these experiments are confounded by incorporation during ISC incorporation; this that can be transmitted to new EBs through mitosis.

We believe that our evidence that EB remain diploid is strong enough to support our conclusion that zfh2-mediated activation is required for downstream processes, including endoreplication.

**2. The authors nicely show elevated p4EBP staining with UAS-zfh2 expression, but the corresponding Western blot in Fig5A and the three panels in FigureS4A are not so convincing. Is it possible to do a pS6K Western in the same condition? By the way, there is a curious observation in the b-actin blot: there are two bands in the UAS Zfh2 and UASRheb, but not in the controls. No I am not asking the authors to resolve the mystery, but if they happen to know why already, it would be nice to share in the figure legend.**

As suggested by reviewer1, we conducted a new series of p4EBP immunostaining to support our conclusion that zfh2 overexpression causes an increase in Tor activity in EBs. We now show that significant levels of p4EBP can be detected both with the esgGal4ts and GBEGal4ts drivers. We also confirm that specificity of the detected signal using UAS-Rheb and UAS-Thor/4EBPRNAi. These results are presented in the revised Figure5A and Figure S4.

Unfortunately, in our hands, no reliable signal can be detected by westernblot from intestinal extracts with the pS6K antibody that we tried.

The extra Actin band in some of our genetic manipulations is a complete mystery to us at this point.

**3. The staining with Zfh2 antibody is faint in Figure S1, but am I correct to say that in the non-DSS treated guts, the staining is more diffuse, almost seems cytoplasmic. With DSS treatment, the nuclear staining becomes more prominent and discernable? This seems more obvious to me in the 18hr panel. It is difficult to draw such a conclusion from the figure presentation. It is also unlikely as in previous studies of Zfh2 expression pattern in the wing disc (Terriente et al, 2008), strong nuclear staining of Zfh2 is evident. However, if the authors do concur with my observation, does it imply that Zfh2 is translocated in the nucleus in after stress? Again, this is just my curiosity.**

It is true that the zfh2 signal appears more diffused in one of our images in Figure S1A (control – 6hours), although it is clearly enriched in the nucleus of several cells. However, this is not evident in other images (of this specific time point, not shown) or at other control time points in the same figure (18h or 40h). In addition, zfh2 appears mostly nuclear in untreated epithelia (see Figure 1A for example). Overall, we found no convincing evidence that zfh2 may have a different subcellular localization during tissue regeneration or under homeostatic conditions. This was an interesting possibility that we explicitly investigated, as the activity of the mammalian homolog ATBF1 can be regulated by cytoplasmic to nuclear shuttling in specific biological contexts. We mention these observations in our discussion on Page24.

**4. It would be very useful to include scale bars in all the confocal images, and include a clear description of what the arrowheads and stars indicate throughout of figure legend. The authors did so for very few panels. One can deduce what they point to, but the readers should not have to.**

We have edited the figures and their legends to incorporate scale bars and better describe the symbols used.

**5. Figure 4 panels do not match the figure legend description. Everything is shifted, 4A in the figure is 4B in the legend, 4B is 4C, 4C is 4A.**

This has been corrected in the revised version

**6. There is no figure 6G (page 14, line 295). Do the authors mean 6B?**

This has been corrected in the revised version
